# Supplementary material for: The nucleolar DExD/H protein Hel66 is involved in ribosome biogenesis in Trypanosoma brucei
Source: Sci Rep. 2021 Sep 15;11:18325. doi: 10.1038/s41598-021-97020-0 (PMC8443567; doi:10.1038/s41598-021-97020-0)
Supplement: Supplementary file 1 — Supplementary Information. [file 41598_2021_97020_MOESM1_ESM.pdf]

SUPPLEMENTARY INFORMATION

The nucleolar DExD/H protein Hel66 is involved in ribosome biogenesis in *Trypanosoma brucei*

Majeed Bakari-Soale[1], Nonso Josephat Ikenga[1], Marion Scheibe[2], Falk Butter[2], Nicola G. Jones[1], Susanne Kramer[1], Markus Engstler[1*]

[1]Department of Cell and Developmental Biology, Biocentre, University of Würzburg, 97074 Würzburg, Germany
[2]Quantitative Proteomics, Institute of Molecular Biology (IMB), 55128 Mainz, Germany
[*]corresponding author: markus.engstler@biozentrum.uni-wuerzburg.de

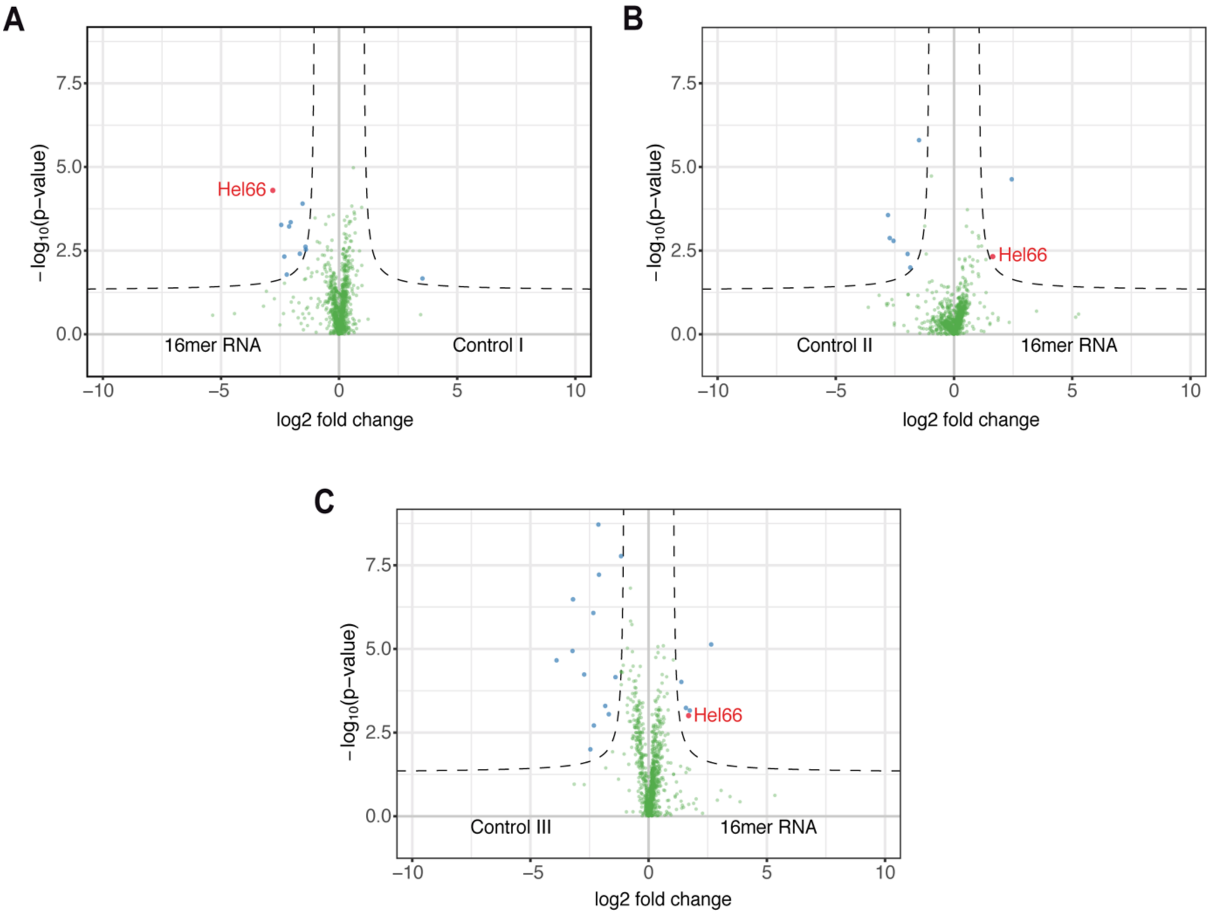

Supplementary Figure S1: Volcano plots showing potential interaction partners of the 16mer motif. The first 188 nucleotides of the VSG121 3´ UTR (following the stop codon) harbouring the 16mer and 8mer motifs were in vitro transcribed and used as bait. (A-C) Triplicate assays using different controls (control I: first 188 nucleotides of the VSG121 3´ UTR with scrambled 16mer and 8mer, control II: reverse complement of control I, control III: first 188 nucleotides of the VSG121 3´ UTR with reverse complement of 16mer).

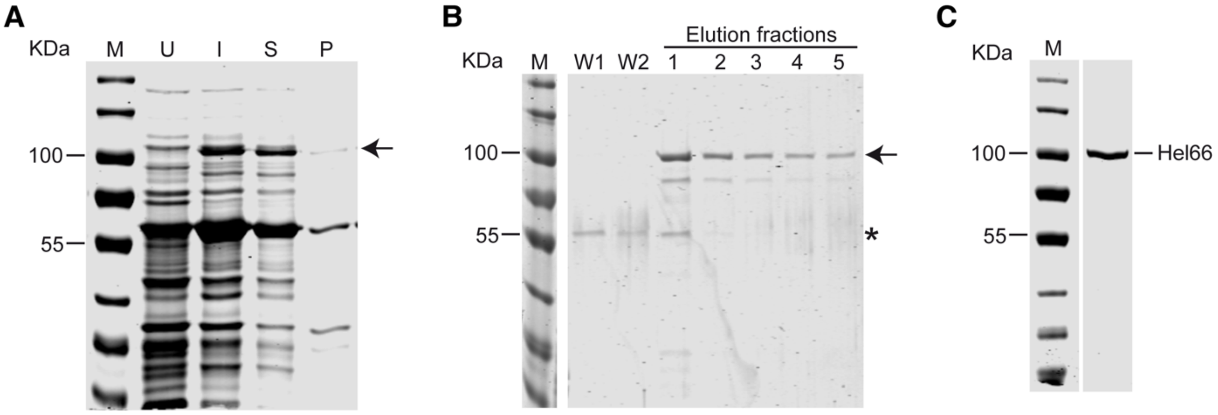

Supplementary Figure S2: Expression and purification of recombinant GST-tagged Hel66 (GST-Hel66). (A) SDS page gel showing GST-Hel66 (arrow) expressed in ArcticExpress *E. coli* cells. M = Marker, U = uninduced, I = induced, S = supernatant (soluble fraction) and P = pellet (insoluble fraction) (B) Purification of GST-Hel66 (arrow) using GST-Gravitrap column. M = Marker, W1= flow-through from first wash, W2 = flow-through from second wash, 1-5 different elution fractions. Asterisk (*) indicates the position of the co-purifying chaperon Cpn60 of 55 kDa molecular weight. (C) Concentrated purified GST-Hel66 protein.

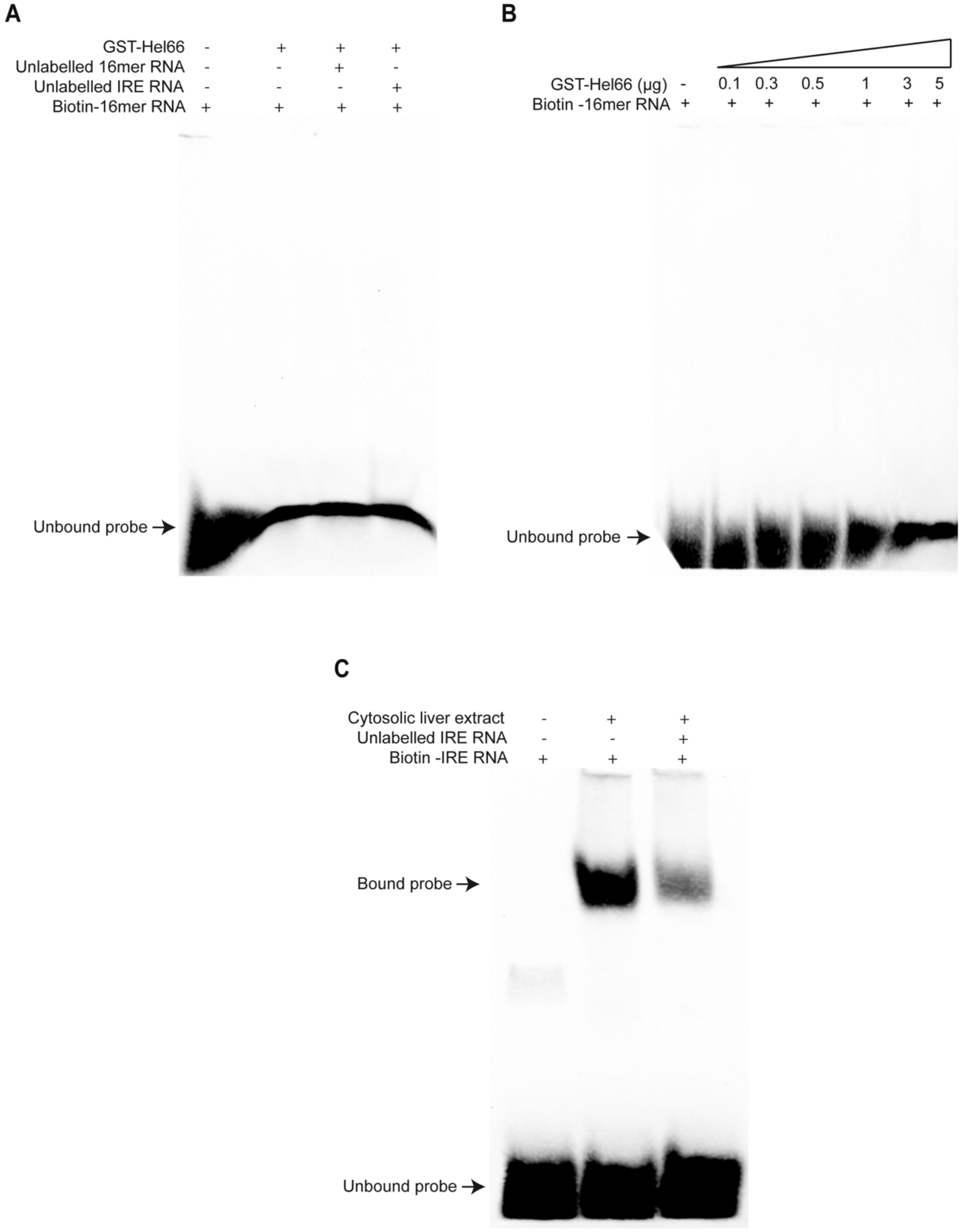

Supplementary Figure S3: The 16mer RNA does not interact with GST-Hel66 (A) REMSA using 16mer RNA and GST-Hel66. 10 nM of biotinylated 16mer RNA was incubated at room temperature with 5 µg of GST-Hel66 in a reaction mix of 20 µl for 30 min. Competition assays were carried out by adding 200-fold excess of either unlabelled 16mer RNA or an unrelated RNA (IRE RNA). (B) REMSA assay with the 16mer RNA and different concentrations of GST-Hel66 protein. 10 nM of biotinylated 16mer RNA was incubated at room temperature with GST-Hel66 in a reaction mix of 20 µl for 30 min. (C) Positive control from the REMSA kit showing functional binding/interaction between IRE (iron-response element) RNA and IRP (iron-response protein).

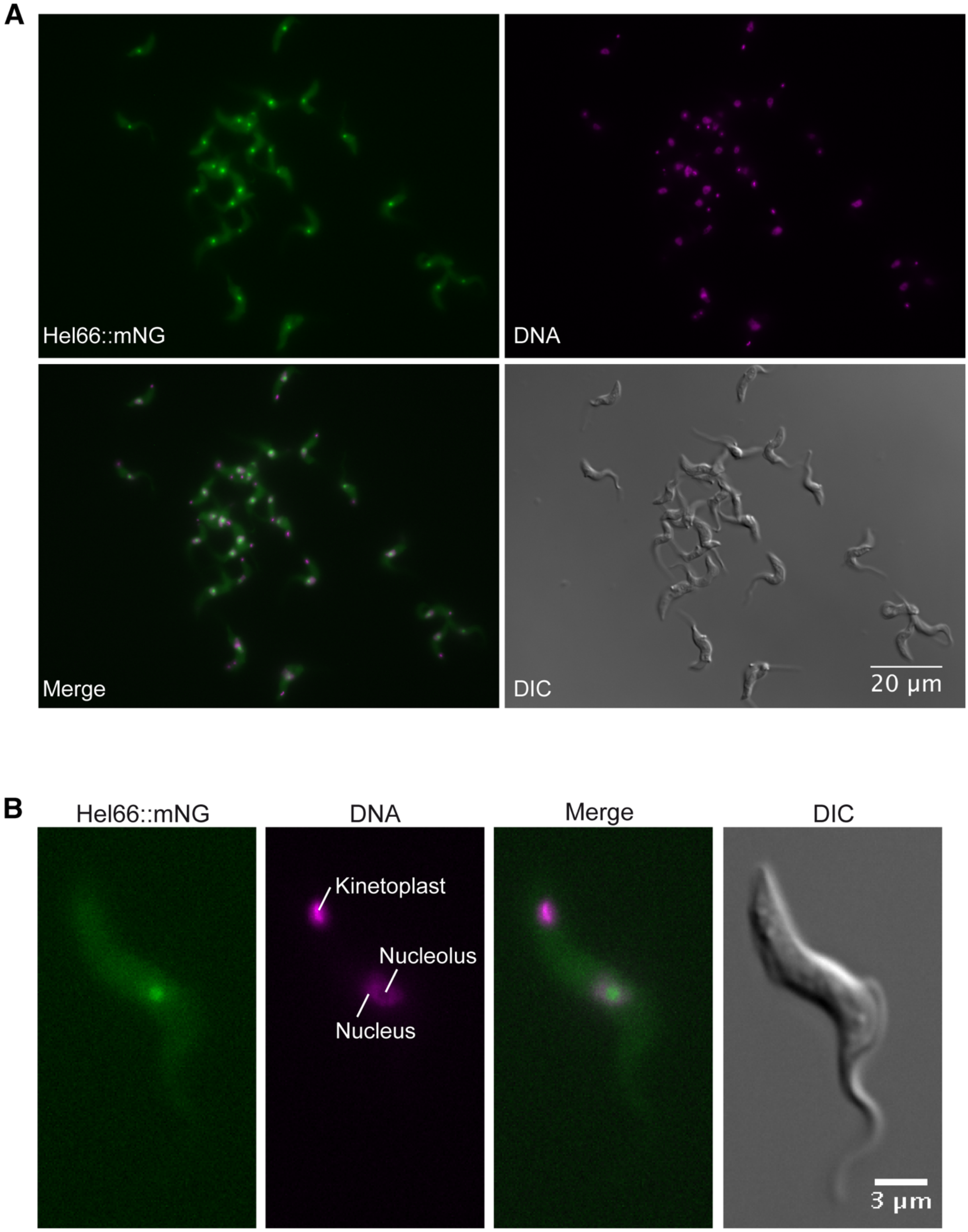

Supplementary Figure S4: Hel66 fused to mNeonGreen localises to the nucleolus in *T. brucei* bloodstream form cells. (A) Localisation of Hel66 in a population of cells. (B) Raw images of one example of a cell showing the nucleolar localisation of Hel66.

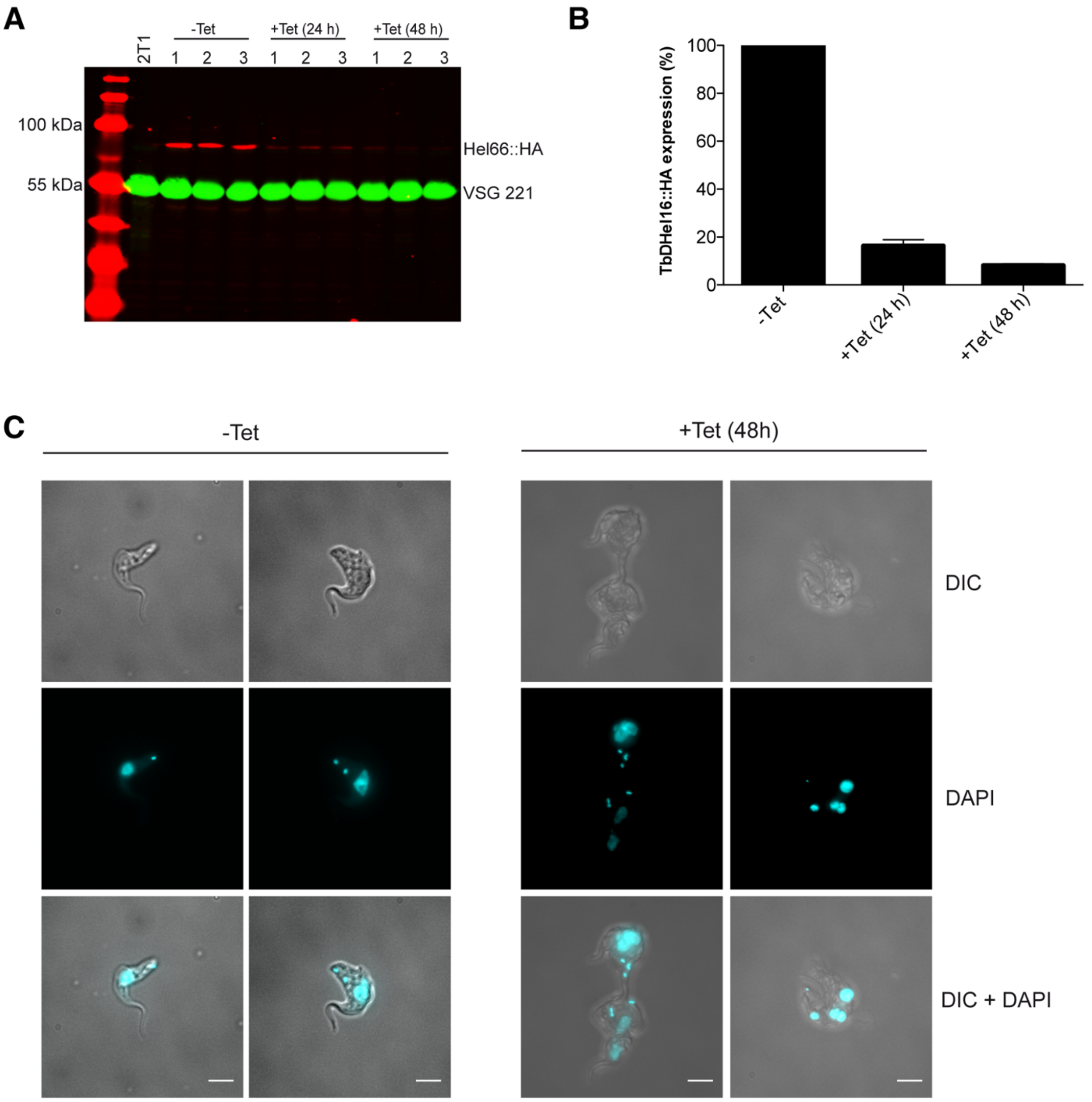

Supplementary Figure S5: RNAi-mediated depletion of Hel66 (A) Western blot showing the protein amounts of Hel66::HA for three independent clonal cell lines over a time following RNAi induction. 2T1 is the parental cell line. VSG221 served as a loading control (B) Quantification of the protein amounts of Hel66::HA. The signal intensity of Hel66::HA was normalised to the VSG221 signal. Average data from the three independent clonal cell lines are shown, with the standard error of the mean (SEM) presented by error bars. (C) Examples of cells with aberrant morphology following RNAi depletion of Hel66 (right); uninduced cells are shown as a control (left). Scale bar = 5µm.

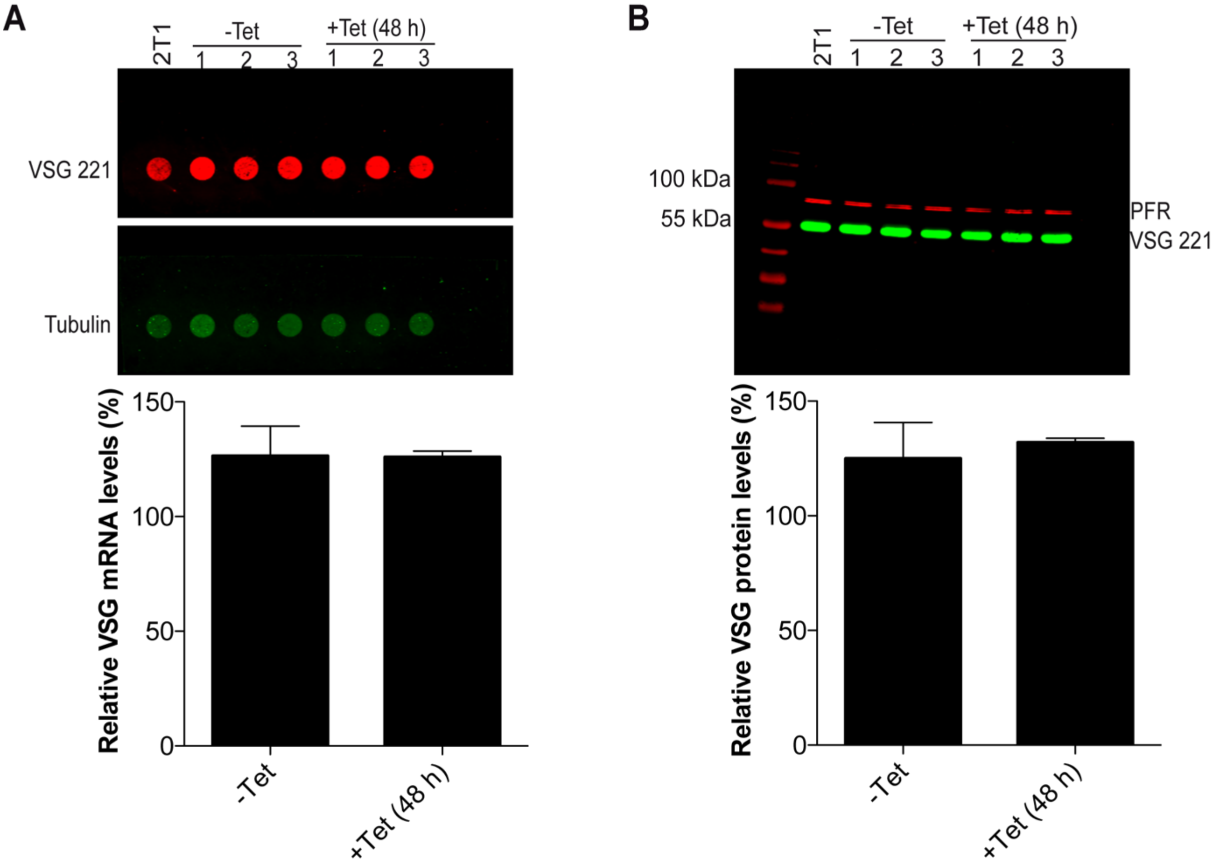

Supplementary Figure S6. *VSG221* mRNA and protein levels upon depletion of Hel66 (A) *VSG221* mRNA levels in uninduced (-Tet) and induced (+Tet 48 h) Hel66-RNAi cells. *Tubulin* mRNA was used as a loading control. Average data of three independent clonal cell lines are shown with error bars representing the standard error of the mean (SEM). (B) VSG221 protein levels in uninduced (-Tet) and induced (+Tet 48 h) Hel66-RNAi cells. A paraflagellar rod protein (PFR) was used as a loading control. Average data of three independent clonal cell lines are shown with error bars representing the standard error of the mean (SEM).

Supplementary Table S1. List of primers and probes used in the study

Name	Sequence (5' – 3´)	Reference
IN3	ggatccATGAACATCTACAGTTGGG	This study
IN4	gaattcTTAATTTTGTGGTGTGACTTCC	This study
MBS37	ggggacaagtttgtacaaaaaagcaggctGACCATCGTGTTTCACCC	This study
MBS38	tattgtgtttcgggagcgacaAGGGAAGTAGAGCCTCGAAC	This study
MBS39	gttcgaggctctacttccctTGTCGCTCCCGAAACACAATA	This study
MBS40	ggggaccactttgtacaagaaagctgggtGTTCATCGTGCAGTTGTAAGCA	This study
VSG221	IRDye 682-CAGCGTAAACAACGCACCC TTCGGTTGGTCGTCTAG	Batram et al., 2014[58]
Tubulin	IRDye 782-ATCAAAGTACACATTGATGCGCTCCAGCTGCAGGTC	Batram et al., 2014[58]
Pre-18S	DY682-TCAAGTGTAAGCGCGTGATCCGCTGTGG	Sakyiama et al., 2013[27] with modifications
ITS2	DY682-ATCACTCACTACACACACGTAT	Umaer et al., 2014[13] with modifications
ITS3	DY682-ACGACAATCACTCACACACACATGGC	Jensen et al., 2003[23] with modifications
16mer	Biotin-UGAUAUAUUUUAACAC	This study

Overhangs are shown in lower case.

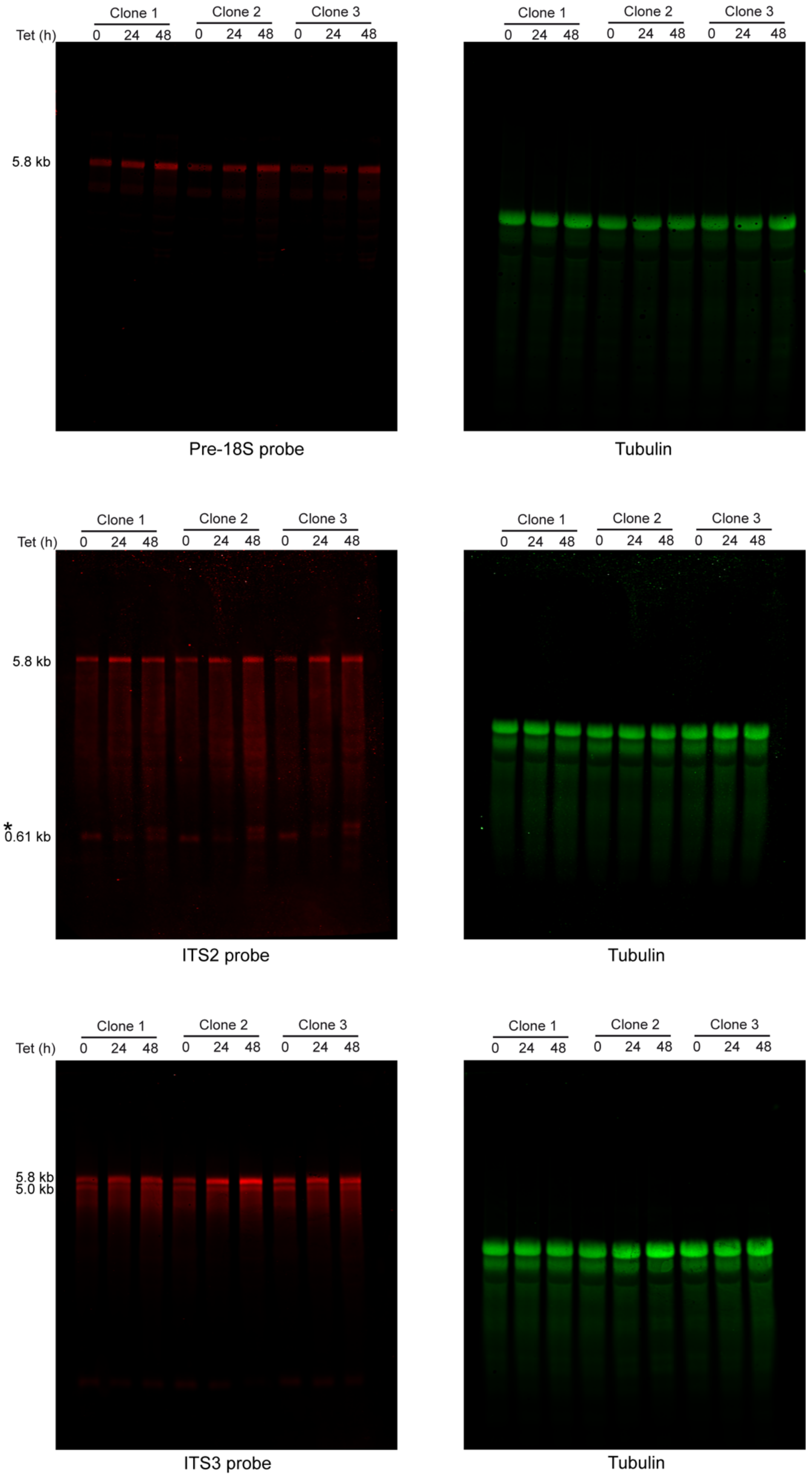

Figure S7. Depletion of Hel66 results in accumulation of rRNA processing intermediates. Images provided here are raw (unprocessed) images of blots in Figure 4.

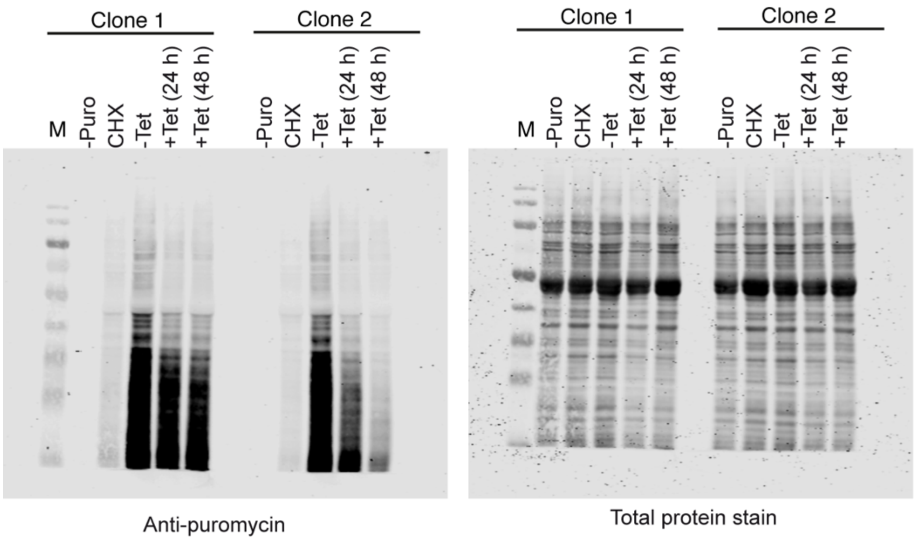

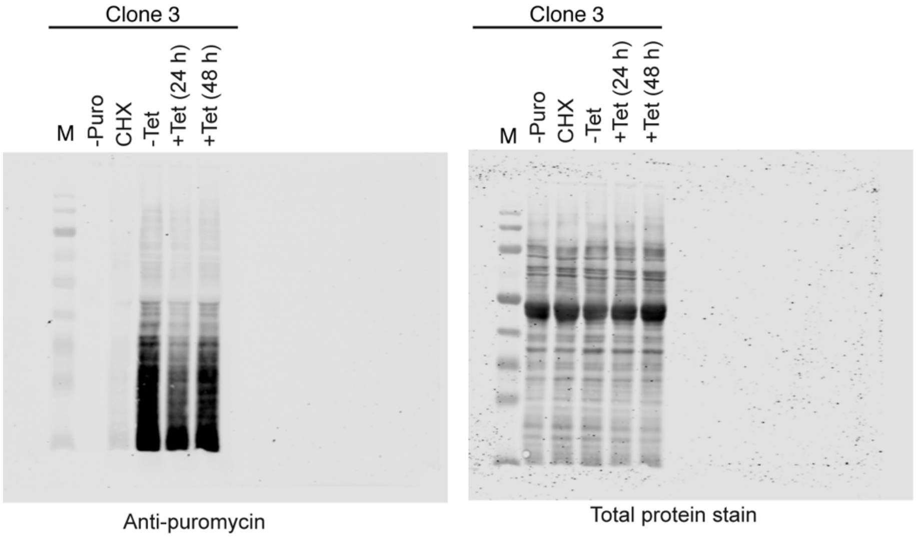

Figure S8. Global translation is decreased upon depletion of Hel66. Images provided here are raw (unprocessed) images of blots in Figure 5. All three clonal cell lines used for the quantification are shown.
